# Feature-Based and String-Based Models for Predicting RNA-Protein Interaction †

**DOI:** 10.3390/molecules23030697

**Published:** 2018-03-19

**Authors:** Donald Adjeroh, Maen Allaga, Jun Tan, Jie Lin, Yue Jiang, Ahmed Abbasi, Xiaobo Zhou

**Affiliations:** 1 Lane Department of Computer Science and Electrical Engineering, West Virginia University, Morgantown, WV 26508, USA; maenallaga@gmail.com (M.A.); jforce716@gmail.com (J.T.); 2 Faculty of Software, Fujian Normal University, Fuzhou 350108, China; linjie891@163.com (J.L.); yueljiang@163.com (Y.J.); 3 McIntire School of Commerce, University of Virginia, Charlottesville, VA 22904, USA; ana6e@comm.virginia.edu; 4 McGovern Medical School, and School of Biomedical Informatics, The University of Texas Health Science Center at Houston (UTHealth), Houston, TX 77030, USA; Xiaobo.Zhou@uth.tmc.edu

**Keywords:** RNA Protein Interaction, RPI, *k*-mers, suffix trees, richness, protein structure, RNA structure

## Abstract

In this work, we study two approaches for the problem of RNA-Protein Interaction (RPI). In the first approach, we use a feature-based technique by combining extracted features from both sequences and secondary structures. The feature-based approach enhanced the prediction accuracy as it included much more available information about the RNA-protein pairs. In the second approach, we apply search algorithms and data structures to extract effective string patterns for prediction of RPI, using both sequence information (protein and RNA sequences), and structure information (protein and RNA secondary structures). This led to different string-based models for predicting interacting RNA-protein pairs. We show results that demonstrate the effectiveness of the proposed approaches, including comparative results against leading state-of-the-art methods.

## 1. Introduction

The interaction between proteins and RNA is known to be an important cellular process. RNA interaction with malfunctioning proteins has been implicated in cell misregulation [1,2], leading to serious diseases, such as cancer, cardiovascular diseases, and neurological disorders [2]. Yet, we still lack a complete understanding of the characteristics of a protein or of an RNA that allow them to interact. Even less is known about those characteristics that play a significant role in the formation of new protein-RNA complexes. Various groups have thus studied interactions for specific pairs of protein and RNA, even without knowing the general properties that facilitate or inhibit such interactions. Starting with experimentally known interacting RNA-protein pairs, computational methods, such as machine learning can be brought to bear on the problem, by attempting to predict possible RNA-protein interactions based on information from the already known interacting RNAs and proteins [3].

The protein or RNA secondary structure describes how the molecules are bound together in a three dimensional space, and therefore can play a crucial role in characterizing the interaction process. The RNA secondary structure describes how some nucleotides in the single stranded RNA are paired to form potentially complicated structures, such as stems, loops, and hairpins. Some methods have been developed to predict RNA secondary structure based on the nucleotide sequences. Thermodynamic methods are the most popular amongst these methods [4,5]. These mainly rely on the notion of free energy, and building secondary structures based on the minimum free energy principle [4]. On the other hand, protein secondary structures describe how the amino acids are positioned in a three dimensional space. Various approaches have been used to describe the protein secondary structure. A popular approach is by using the dihedral angles (φ and ψ) between the amino acids. Dihedral angles define the angles of rotation between two planes, in this case, the planes are defined by the bonds between three adjacent amino acids [6]. Ramachandran codes are derived from Ramachandran plots (which are 2D charts describing the distribution of the dihedral angles), by reducing information in the plot to some clusters [6,7,8]. Another approach is the protein blocks, which describe protein secondary structures based on the folds formed by five consecutive amino acids and then clustering these folds [9]. 

Earlier methods for RPI prediction focused on sequence data, building features based on only sequence information for the RNA and protein individually, or combining both sequences to extract representative features [3]. Recently, secondary structures were shown to be important in the interaction process, and are now being included in the prediction [10]. In this work, we consider different representations for proteins and RNAs. We consider sequence and structural information for both protein and RNA. For the sequences, we use the traditional 4-letter alphabet for RNA (A, C, G, U), but a 7-letter reduced alphabet for protein. For structures, we use the Ramachandran codes for protein structure representation. These have been used in previous work on studying protein structures [6,7]. However, this work represents the first time Ramachandran codes are being used for protein-RNA interaction studies. RNA secondary structures have been used earlier in RNA alignment and protein-RNA interaction prediction. In this work, we consider string-based representations for both the RNA secondary structure, and the protein secondary structure, in addition to the traditional protein and RNA sequences.

Armed with these representations, we build two different prediction models for RPI prediction. The first extracts features from protein sequences and protein structures and combines these with features extracted from RNA sequences and RNA structures. The idea is to combine all the information about each protein and RNA individually, and then trying to use the integrated information to address the RPI prediction problem. The second model is a string-based approach. Here, we build a feature space based on *k*-grams (i.e., *k*-length substrings, also called *k*-mers in the biology literature). We analyze the sequences of protein-RNA pairs to determine the *k*-mers that tend to appear in interacting pairs, and those that are often found in non-interacting pairs. We then use these *k*-mer pairs as our descriptors (feature set) for predicting RNA-protein interaction. Under the string-based approach, we apply the *k*-mer description approach on both sequence-based and structure-based information, after encoding the structural information as strings. 

The remainder of this paper is organized as follows: Section 2 provides some background, and discusses previous work on predicting RNA-protein interaction. Section 3 describes the feature-based model, while Section 4 presents the proposed string-based approach. Section 5 presents the results, including comparison with state-of-the-art methods. Section 6 concludes the paper.

## 2. Background and Related Work 

The importance of RNA-protein interaction comes from the key role it plays in regulating cellular processes. Researchers have shown interest in RNA-protein interactions primarily driven by the need to understand how cells work, including cell localization and other fundamental processes [10]. Studies have shown that some cases of RNA-protein interaction are related to some important diseases, such as cancer, Alzheimer’s disease, fragile X syndrome, and cardiovascular diseases [2]. Clearly, the problem of prediction is closely related to the issue of representing the RNAs and proteins. Success in identifying and extracting the information that is most relevant to protein-RNA interaction will no doubt lead to improved computational prediction of such interactions. 

Methods for addressing the RNA-Protein Interaction (RPI) problem can be traced to those that have been used to study the related problem of protein-protein interaction (PPI). The Protein-Protein interaction problem is well studied due to the importance of proteins in all cell processes, including coding and decoding genes. Shen et al. [11] were among the first to predict protein-protein interaction using only sequence information. They used a simplified 7-symbol alphabet to represent protein sequences and built a Support Vector Machine (SVM) prediction model using a high quality dataset containing 16,443 experimentally validated entries. Based on the identified interactions, they build a protein interaction network that shows the relationships and connections between different proteins [11]. For other related work on protein-protein interaction see [12,13,14].

Muppirala et al. [3] explored RNA and protein sequences separately. They build a 599-dimensional feature space, with 343 features extracted from protein and 256 features from RNA. Similar to Shen et al. [11], the 343 protein features were extracted by first considering the 7-class reduced protein alphabet, whereby the 20 amino-acids are clustered into seven groups based on their dipole moments and their side-chain volumes. To conserve locality information, the notion of triads was used to extend the feature space to 7 × 7 × 7 features for protein, and 4 × 4 × 4 features for RNA. Two classification models (SVM and Random Forests (RF)) were deployed to build the prediction scheme. The models were trained using two datasets, namely, RPI369 and RPI2241. The Random Forest model trained with RPI2241 obtained the most accurate results among other trained models, achieving 89.6% in accuracy, with 0.89 and 0.90 for precision and recall, respectively.

In [15], Wang et al. applied *k*-mer approach by finding the pairs of protein amino acids and RNA nucleotides that tends to appear together. They worked with a reduced protein alphabet, whereby the 20 amino acids were grouped based on their charge and polarity. The new alphabet consisted of 4 groups representing the 20 amino acids. Then, they considered protein 4-mers and RNA 3-mers. This allowed them to preserve some locality information, which was indicated to have high impact on the prediction process. The feature space consisted of a 4096-dimensional space (4^3^ features for protein, and 4^3^ features for RNA). This high dimensional space requires relatively large datasets for training. Thus, they selected only 500 features that showed the highest impact on the prediction, and adopted the naive-Bayes classifier as the basic classification method. They tested the method on different datasets, including RPI369, RPI2241 and NPInter. The accuracy achieved on these three datasets were 75%, 74% and 77.6%, respectively.

RPI-Pred [10] was developed by Suresh et al. to predict the interaction between non-coding RNA and proteins. They included information from both sequences and secondary structures, building a feature-vector with 132 features. Here, they used 20 features to describe the RNA considering four different nucleotides and five secondary structure elements including stem, hairpin, loop, and bulge. They used 112 features to characterize proteins, following the reduced 7-class alphabet for amino acids, and the extracted 16 protein blocks [9]. They introduced a new dataset, namely, RPI1807 and used this for training. They tested their method using three datasets, namely, RPI369, RPI2241 and NPInter. Using SVM as the prediction scheme, they achieved an accuracy of 92%, 84% and 86.9% using RPI369, RPI2241 and NPInter, respectively.

Lu et al. [16] followed a different approach in predicting RNA-protein interaction. The core difference in their work is in how they extracted their features. RNA secondary structures were predicted using Vienna RNA package [4]. Additionally, they used hydrogen bonding information, then using the Fourier transform they extracted a feature set for both RNA and protein to form the feature vector for each RNA and protein. They included the first ten terms of the Fourier series for each information type. They built a training dataset containing 649 non-redundant protein-RNA pairs (322 of these were interacting pairs, and the remaining 327 were non-interactive pairs). They trained a scoring matrix which gives a score for each protein-RNA pair. Based on the assigned score, they predict the interaction between protein and RNA. The method achieved a 77% accuracy on the NPInter dataset.

Following recent success of convolutional neural networks in different applications, deep learning approaches have been applied to various related problems in biomedicine. Examples here include, in predicting the sequence specificity for DNA-RNA binding proteins (e.g., DeepBind [17] and DeeperBind [18]), mining RNA-protein binding motifs (e.g., iDeep [19]), or in studying the functional effects of non-coding variants (e.g., DeepSEA [20]). See [21,22] for recent reviews on the use of deep leaning in biomedical applications. In particular, Pan et al. [23] studied the use of deep learning on the problem of RNA-protein interaction prediction. They introduced Interaction Pattern Miner (IPMiner), which used deep leaning models to predict potential interaction between non-coding RNA and proteins. They focused on the use of sequence-only information, and investigated the impact of various deep learning architectures on the prediction performance. They also introduced the RPI488 RNA-protein interaction dataset, which is distinct in that the dataset contained protein-RNA pairs involving mainly long non-coding RNAs (lncRNAs). The problem of RNA-protein interaction has also been considered from the viewpoint of complete structural representations. Zhang et al. [24] developed a deep learning model to define the preferences of RNA Binding Protein (RBP) structural representations. They used information from predicted RNA tertiary structures to study the problem of RPI. This helped them to define a 3D representation for RPI complexes, which were then used to describe the binding preferences. 

For both RNA and protein, their sequences and secondary structures can each be expressed in the form of strings [25,26]. In this work, we take advantage of efficient string algorithms and data structures to extract discriminative feature sets and build prediction models that can predict RNA-protein interaction. The other contribution is the development of a prediction model built on Ramachandran codes for protein structures, and the use of enhanced descriptors of RNA secondary structure elements, based on their lengths. In this work, we used both SVM and RF to explore the influence of classification schemes on the prediction performance.

## 3. Feature-Based Model 

In this work, we will use information from both sequences and secondary structures. This applies to both the feature-based approach discussed in this section, and the string-based approach we will introduce in the next section. Features are the attributes of the RNA and proteins that will be used in the prediction. We will build our feature space based on the different elements captured from the RNA/protein sequences and secondary structures. In this section, we will introduce our RNA and protein representations. Then we will define our feature space, and describe the classification approach. 

### 3.1. Representing RNAs 

**Figure 1:**
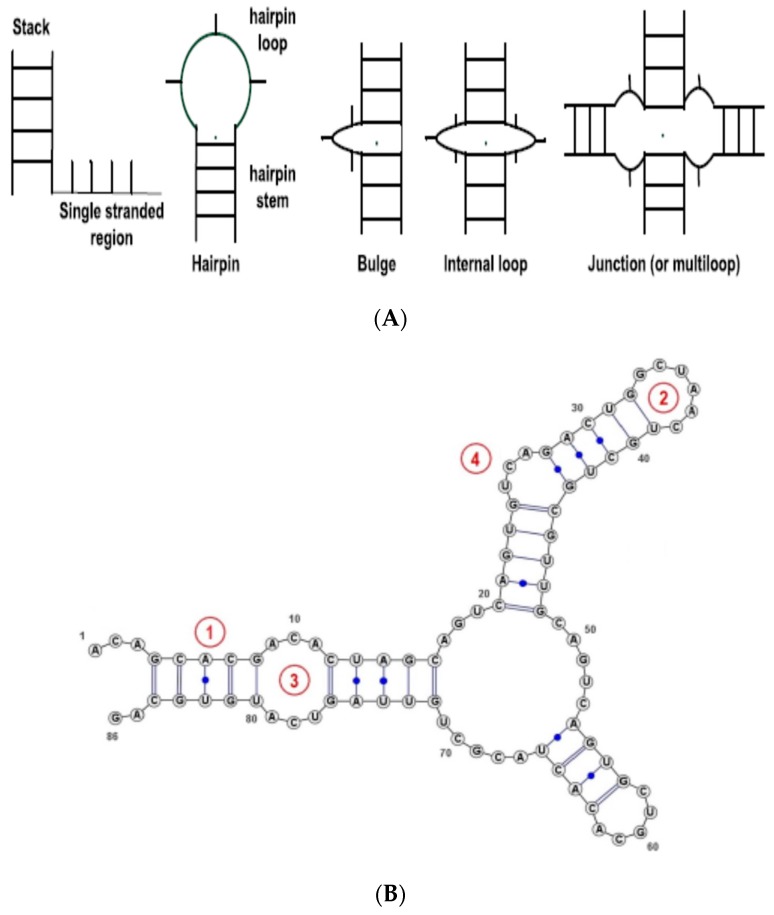
RNA secondary structure elements (SSEs). (**A**) basic types of SSE types, from [25]; (**B**): Example of a short RNA sequence with annotated SSEs: (1) Stem, (2) Loop, (3) Internal loop, (4) Bulge.

For RNA sequences, although a nucleotide can be any of A, U, C and G nucleotides, some RNAs are not completely known and their sequences include special characters, such as ‘X’ at some positions denoting that the nucleotide at this position is unknown. Thus, in this work the alphabet for RNA sequences is extended to five characters (A, U, C, G, X). This alphabet will be used to encode RNA sequences in this work. We also use information from the RNA secondary structure. In this work, when the RNA structure is unknown, we will use RNAFold (part of the Vienna RNA suite of programs [4]) to predict the structure (using RNAfold default parameters). The method implements a free energy model to predict the secondary structure for a given RNA sequence. To describe the RNA secondary structure, we use the RNA secondary structure elements (SSE)—see Figure 1. For simplicity, we consider only the basic types, namely, Stem (or stack), Loop, Internal Loop, Bulges, and Others (non-classified, such as single strands). The RNA secondary structure is thus represented as a string—sequence of basic SSEs. 

**Figure 2:**
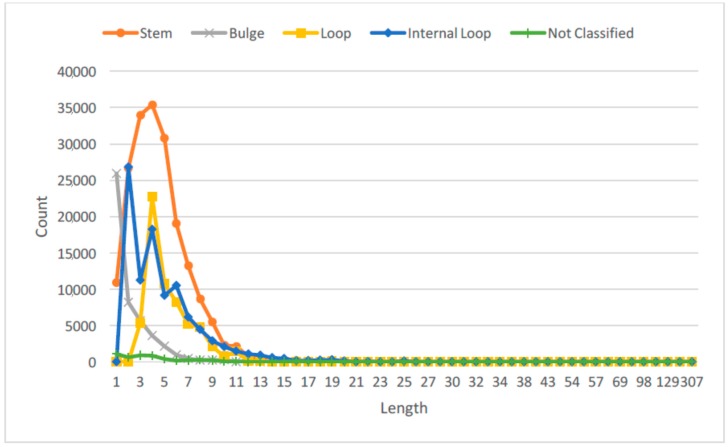
Distribution of lengths for RNA secondary structure elements (SSE). Not classified denotes single stranded regions, and others that are not one of the four main SSE types.

Recent studies have also suggested that RNAs with different lengths or sizes can have different functional roles [27,28]. Hence, we studied the distribution of the lengths for the RNA SSEs, and thus obtained three different length categories for each secondary structure element: long, medium and short. Figure 2 shows the probability distribution of the RNA SSE lengths using the 1078 RNA sequences from the RPI1807 dataset. 

This provides more important information about the RNA secondary structure elements than just considering only the secondary structure elements directly. We can observe that by considering the lengths of different SSEs over the entire RNA sequence, we can capture different RNA configurations, for instance, long stem, large loop; short stem, large loop; or short stem, small loop; large stem, small loop; etc. See figures in [28] for some examples. For each type of SSE, the cutoff for length classes was obtained by using the empirical distribution shown in Figure 2, such that each length type will have about the same probability of being observed in the dataset. Table 1 shows the length thresholds used for the three length classes over the different SSE types.

### 3.2. Representing Proteins

Given the chemical similarity between amino acids, we can partition amino acids into different groups and use the groups, rather than the individual amino acids, to represent protein sequences. Grouping the amino acids can be done based on various criteria. In this work, we follow [3,10], where amino acids were classified based on their dipole moments and their side chain volumes. This way the original 20 amino acids are classified into seven groups: I: {Ala, Gly, Val}, II: {Ile, Leu, Phe, Pro}, III: {Tyr, Met, Thr, Ser}, IV: {His, Asn, Gln, Trp}, V: {Arg, Lys}, VI: {Asp, Glu} and VII: {Cys}. The protein secondary structure is defined by the positions of consecutive molecules in 3D space. The positions can be described by the dihedral angles between each three consecutive molecules, denoted omega (ω), phi (φ) and psi (ψ). Due to the limitation in ω angle, usually only φ and ψ angles are considered in describing the protein secondary structure. Ramachandran et al. [29] studied the relationship between φ and ψ and presented 2D plots (now called Ramachandran plots), showing the density of the joint occurrences of these angles. This density can be used to derive protein secondary structure representations by clustering the values from the Ramachandran plot, then replacing the values of (φ, ψ) pairs by their cluster representative. To represent protein structure, Suresh et al. [10] used 16-character protein blocks. In this work, we will follow the approach in [7], and thus use a 7-symbol alphabet representing seven clusters from the Ramachandran plot. See also [6]. Hence, the protein representation used will consist of a 7-symbol alphabet for amino acid groups (for the sequence), and another 7-symbol alphabet from the Ramachandran codes (for the protein secondary structure).

### 3.3. Feature Space and Classification

Given the representations for the sequences and secondary structures for RNA and proteins, we can now construct our feature space. For RNA sequences, we use the 5-symbol alphabet (A, U, C, G, X) to represent the nucleotides. Also, each nucleotide will belong to one of the 5 secondary structure elements (SSEs) as described earlier. Then, each secondary structure element can have three different length classes. This results in a 5 × 5 × 3 feature space, or a total of 75 features representing an RNA. Protein sequences are reduced to seven groups resulting in a 7-symbol alphabet. Protein secondary structures are also represented using 7 different Ramachandran code clusters for each position. Thus, the protein representation requires a 7 × 7 feature space, or 49 features. The power of this representation comes from the fact that it includes all the available information using a small number of features. This is crucial in building the prediction model in reasonable time and space, and to ensure accuracy in the prediction. 

Building the feature vector and finding the best representation for the prediction model is just the first step in RNA-protein interaction prediction. The next step is to decide what prediction scheme should be used. The RNA-protein interaction prediction problem is to determine whether or not a given pair of protein and RNA will interact. Essentially, this can be viewed as a supervised learning problem, specifically, a binary classification problem. Different algorithms have been developed to solve classification problems. However, considering the protein-RNA interaction problem, Support Vector Machines (SVM) and Random Forest (RF) proved to be among the best classification methods in prior work. Therefore, we will consider these two methods and compare the performance of both classifiers using our approaches. 

## 4. String-Based Approach 

Although the feature-based approach is powerful and contains a lot of information about RNA and protein, it does disregard important local information. This local information could be important in the prediction process. When an RNA bonds to a protein, it is not just an amino acid and a nucleotide that are involved, but a set of neighboring nucleotides against a set of amino acids. Furthermore, the secondary structures should be compatible to allow RNA-protein bonding. These observations suggest that we could consider small portions or *k*-grams (*k*-mers) of sequences and structures when building the feature vector rather than looking for individual molecules. This will typically lead to a very large dimensional feature space. Consider for instance, 5-mer strings under a 75-symbol alphabet (using the RNA representation presented earlier). This means we have more than 75^5^ ≈ 2 × 10^9^ possible different 5-mers to consider for RNA only, besides the 49^5^ ≈ 2 × 10^8^ 5-mers for proteins. This is a huge feature space that will be very difficult to handle with current computational limitations. Thus, we need to reduce the feature space dimensionality. A quick observation is that this feature space will be very sparse, as most combinations of the RNA and protein symbols will not occur in practice.

Reducing feature space dimensionality means we need to carefully select some *k*-mers and drop the rest. Not all *k*-mer strings hold the same amount of information with respect to possible interaction between the protein and RNA. Thus, to enhance prediction accuracy, we need to identify the *k*-mers that have the most influence on the prediction of interacting or non-interacting RNA-protein pairs. Hence, we look for the *k*-mer strings that appear mostly in the positive pairs and those that appear mostly in the negative pairs, and then construct a feature vector based on these.

### 4.1. Suffix Trees for Protein and RNA Strings

The naive approach to find the most occurring substrings is to count each of them within the dataset. The running time for this approach will be *O*(*nk*) for each *k*-mer, where *n* is the total number of all symbols in the dataset. This means we need to hold a large dictionary of all possible substrings, the size of this dictionary would be in *O*(*αk*), where α is the alphabet size (in our case, could have α = 75 for RNA and α = 49 for protein). We need to find a better approach to study the distribution of *k*-mers, i.e., a memory- and time-efficient method to find the *k*-mers that contribute most in the interaction process. One way will be to go over the database to first determine all the *k*-mers that actually occurred, and then use standard linear-time pattern matching algorithms to determine their respective number of occurrences. Overall, this will be *O*(*kn*^2^) time worst case. 

A better approach will be to use suffix trees and suffix arrays [26,30,31] to provide a better tool to find the most occurring substrings within positive pairs and negative pairs. The suffix tree requires an *O*(*n*) time and space for construction. After construction, we can traverse the *O*(*n*) nodes of the suffix tree to determine the occurrence counts of all substrings in *O*(*n*) time. Thus, this is the time required to count all the *k*-mers in the string, independent of *k*. The power in using the suffix tree to find the distribution of *k*-mers is that we don’t need to maintain a large dictionary. We built suffix trees counting occurrences of each substrings of length 2 to 5 for RNA sequence and secondary structure, and protein sequence and secondary structure. That is, we constructed four suffix trees, one for each type of string representation we used, namely: RNA sequence, RNA secondary structure elements represented as strings, protein sequence, and protein secondary structure represented as a sequence of Ramachandran codes.

### 4.2. Richness for Protein and RNA Substrings

In general, the *k*-mers that tend to occur more in positive pairs (i.e., interacting RNA-protein pairs) would provide more information in deciding on a positive pair than other *k*-mers that appeared equally in both positive and negative pairs. Similarly for *k*-mers that appeared more in negative pairs. With this observation, we should look for more than just occurrence counts. The richness could be a better measure of the contribution of a *k*-mer to the interaction between RNA-protein pairs. Given a *k*-mer, say *β*, let *γ*(*β*) = #occurrences of *β* in the positive pairs, and let *λ*(*β*) = #occurrences of *β* in the negative pairs. The *k*-mer richness is simply defined as:

$$R\left(\beta \right)=\frac{\gamma \left(\beta \right)+1}{\lambda \left(\beta \right)+1}$$

Thus, *k*-mers with richness greater than 1 appear more in positive pairs (positive *k*-mers), while a richness value near zero means the *k*-mer appeared mostly in the negative pairs (negative *k*-mer). Richness values close to 1 are associated with *k*-mers that appear equally in both positive and negative pairs, hence they provide less discrimination ability between interacting and non-interacting pairs. 

**Figure 3:**
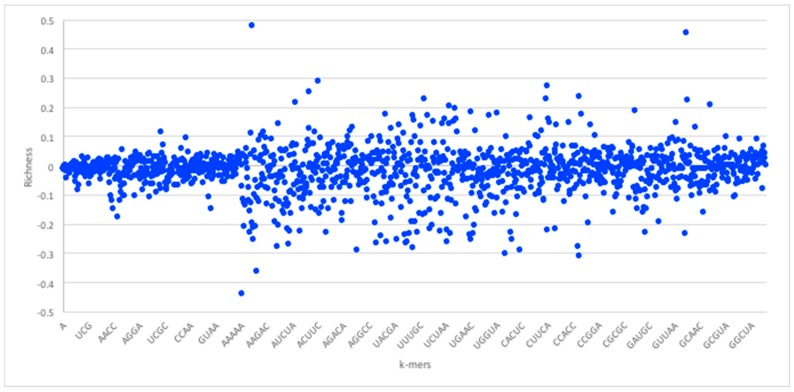
RNA sequence *k*-mer richness (log values) using the RPI1807 dataset. RNA sequence symbols are taken from the 5-symbol alphabet (A, U, C, G X). Not all *k*-mers are shown.

**Figure 4:**
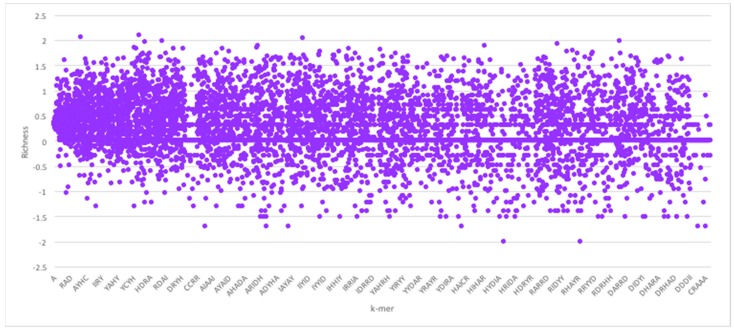
Protein sequence *k*-mer richness (log values) using the RPI1807 dataset. Protein sequence symbols are taken from the 7-symbol alphabet (A, I, Y, H, R, D, C). Not all *k*-mers are shown.

Given the foregoing, we now construct four suffix trees for positive pairs in the dataset, and another four suffix trees for the negative pairs. We extracted pairs that appeared only in positive pairs or only in negative pairs as they hold the most discriminative information for interaction prediction. To provide some perspective on the feature space generated, we used the RPI1807 dataset (see Section 5.1 on datasets), to build suffix trees of depth five and computed the distributions for *k*-mers up to length 5. RNA 4-mers appeared for as many as 18,020 times in positive pairs and 16,894 in negative pairs, while for 5-mers, the number drops to 3700 appearances in positive pairs and 3494 negative pairs. Obviously, the numbers drop as the *k*-mer length increases. Protein 4-mers occurred about 1134 times in positive pairs, and 443 times in negative pairs. As mentioned, the direct *k*-mer count distribution may not be the best way to capture the information carried by the *k*-mers. Thus, we considered the richness as the main factor to compare *k*-mers and build the feature vector. Figure 3 shows the *k*-mer richness (log scale) for RNA sequences, using the RPI1807 dataset above. In this chart, positive log values imply *k*-mers that appear more in the positive pairs, while negative values correspond to those that appear more in the negative pairs. Thus, a simple threshold can be used to select the positive *k*-mers and the negative *k*-mers, while avoiding those that are less discriminative (those close to 0). Figure 4 shows the corresponding *k*-mer richness plot for protein sequences. 

### 4.3. String-Based Models 

Based on the foregoing, we then consider string-based prediction models that combine the extracted RNA and protein *k*-mers. Our models consider different combinations of the identified *k*-mers that pass the richness threshold. We consider five models based on these combinations, viz: QQ: RNA sequence *k*-mers in combination with protein sequence *k*-mers; SS: RNA secondary structure *k*-mers and protein secondary structure *k*-mers, QS: RNA sequence *k*-mers and protein secondary structure *k*-mers, and SQ: RNA secondary structure *k*-mers and protein sequence *k*-mers. The final model (QSQS) combines *k*-mers from the first four models, thus exploiting info from RNA sequence, and RNA structure, protein sequence, and protein structure. We have included the Top-100 richness *k*-mers for each of the four string-based models as Supplementary Materials.

## 5. Experiments and Results 

### 5.1. Datasets and Setup

The Protein Data Bank (PDB), Nucleic Acid Database (NDB) and Protein-RNA Interface Database (PRIDB) [32] are widely known databases that provide Protein-RNA complexes which can be parsed to obtain a dataset with positive pairs, and negative pairs. Suresh et al. [10] parsed NDB and PDB to build their dataset, based on atomic interaction between Protein and RNA pairs. That is, they used the atomic distance between RNA and protein sequences extracted from RNA-protein complexes. Using a threshold on the atomic distance, they were able to separate strongly interacting pairs from weakly interacting pairs. Thus, RNA-protein pairs with strong interaction are considered positive pairs, and pairs with weak interaction are considered negative pairs. The dataset they obtained was used to build classifiers which performed very well on other datasets. We call this dataset RPI1807. It has 1807 positive pairs and 1436 negative pairs, for a total of 3243 RNA-protein pairs. RPI369 and RPI2241 are two non-redundant RNA-protein interaction datasets [3]. RPI369 contains 369 RNA-protein pairs, while RPI2241 contains 2241 RNA-protein pairs. Muppirala et al. [3]. extracted RPI369 and RPI2241 from PRIDB. PRIDB is a database that contains RNA-protein complexes extracted from PDB [32]. RPI2241 contains rRNA, ncRNA or mRNA. RPI369 was extracted from RPI2241. It contains only non-ribosomal complexes [3].

In this work, we used the RPI1807 dataset [10] to train our learning models, and to tune parameters. The dataset has 1807 positive pairs (1807 protein and 1078 RNA chains), and 1436 negative pairs (including 1436 protein and 493 RNA chains). Then, we test our methods on other datasets, and compare our results against previous work. Classification was performed using both SVM [33] and RF [34], using Weka [35], version 3.6.13. We used the RPI1807 dataset from [10] to construct our models, and set parameters. We set the parameters for the classification schemes based on empirical observations using the training dataset. SVM parameters were set as C = 2^5^, γ = 2^−7^ for the string-based models, and as C = 2^7^, γ = 2^−11^ for the feature-based model. For RF, the number of decision trees was set to 200, for both string-based and feature-based models. Then, we evaluated the models on the RPI369 and RPI2241 datasets reported in [3]. Both were obtained from the PRIDB dataset of RNA-protein complexes [32] extracted from the protein databank (PDB). RPI2241 includes complexes with rRNA, ncRNA and mRNA, and this is more challenging. We also tested on RPI488, the lncRNA-protein interaction dataset recently introduced in [23].

### 5.2. Performance Measurement

We evaluated our approaches using 10-fold cross-validation. To measure the performance, we used precision (PRE), recall (REC), accuracy (ACC), and F-measure (FSC), viz: PRE = TP/(TP + FP), REC = TP/(TP + FN), ACC = (TP + TN)/(TP + TN + FP + FN), FSC = 2 × (PRE × REC)/(PRE + REC), where, TP is true positive (the count of correctly classified positive pairs), FP is false positive (the count of wrongly classified positive pairs), TN is true negative (count of correctly classified negative pairs), and FN is false negative (count of wrongly classified negative pairs). We also computed the area under the curve (AUC) (with values in [0 1], with 1 indicating perfect prediction).

### 5.3. Results

We tested our methods against different datasets from previous studies to compare our results with previous work. We tested on RPI369 and RPI2241 extracted by Muppirala et al. [3], and also on RPI488 dataset introduced by Pan et al. [23]. We applied two different prediction methods, SVM and RF. In previous work, SVM was reported to perform better than RF (e.g., RPI-Pred [10]), while in some others RF outperformed SVM. (e.g., [3]). Thus, we applied both schemes to see their performance under our framework. 

Table 2 shows the results of our proposed feature-based and string-based methods on the RPI1807 dataset, using both SVM and RF prediction schemes. For the feature-based method, RF outperformed SVM, scoring 94.68% in accuracy and 0.94 in precision, recall and F-measure. The difference between SVM and RF was even more significant using the string-based approach, with RF doing much better with accuracy of 93.35%, and 0.93 in precision, recall and F-measure. Thus, subsequent results are reported for RF classification method.

The string-based approach used a different approach from the feature-based approach, especially with respect to feature vector extraction and representation. We tested *k*-mers extracted from different combinations of RNA sequence, secondary structure, and protein sequence and secondary structures. Table 3 shows the results of our proposed string-based models. Columns 2 and 3 show results for the QSQS model, using both SVM and RF. Clearly, RF is doing much better than SVM using our approach. Thus, subsequent results in this work are reported only for the Random Forest classifier.

As expected, the QSQS model achieved the best result. This is due to its use of more detailed information from both structure and sequence. The results of using only sequences (QQ model) were very close to using all available info. This could be due to the fact that secondary structure is determined by the sequence. Interestingly, using *k*-mers from only the secondary structures (SS model) led to a significant performance drop (ACC = 66.98%). The table shows that sequences provide a key information for RPI prediction. Though secondary structures can help when combined with sequences, they lack precision when used independently.

### 5.4. Impact of Sequence Lengths and SSE Lengths

Given the use of SSE lengths in our approach, we decided to investigate the impact of sequence lengths and SSE sizes on RNA-protein interaction. This can be investigated on three perspectives: (1) impact of the size/length of different SSEs on interaction between a given RNA-protein pair; (2) impact of protein sequence and RNA sequence lengths on their potential to interact; and (3) impact of sequence lengths on the predictability or otherwise of RPI, if any. For the first view, using the RPI807 dataset we analyzed the potential impact of SSE lengths on the ability of a given RNA-protein pair to interact. Table 4 shows the potential influence of SSE types and SSE lengths on the interaction ability for a given RNA-protein sequence pair. The rows in red color show the RNA SSE types (as defined by the specific type of SSE and its length) that tend to appear more in the RNA sequences found in the negative RNA-protein pairs (i.e., those that do not interact), while the green rows show RNA SSE types that tend to appear more on RNA sequences involved in the positive pairs. The column labeled “Dominant” indicates which type of RNA-protein pair where the given RNA SSE type tends to occur most. The table shows that each of the basic RNA SSE types (Stem, Loop, Internal Loop of Bulge) can appear in either the positive or negative RNA-protein pairs. However, when we consider their sizes, we can more easily see those that tend to dominate in positive (interacting) or negative (non-interacting) RNA-protein pairs.

**Figure 5:**
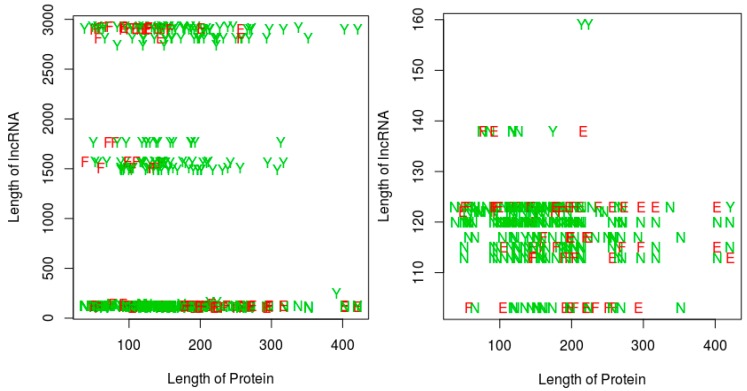
Impact of RNA and protein sequence lengths on potential for RNA protein interaction, and on the ability of an RPI prediction method. Results are shown for the RPI488 dataset. Left: all sequence lengths in the dataset; Right: zooming in on RNA sequences of length 200 nts or less on the Left panel. Legend: Y: interacting pair, correctly predicted; N: non-interacting pair correctly predicted as non-interacting; E: Interacting pair incorrectly classified; F: non-interacting pair incorrectly classified as interacting. Results are based on the QQ string-based model, proposed in this work.

For the last two perspectives, we focus on our proposed RPI prediction method, and thus essentially investigate whether the sequence lengths (protein and/or RNA) have any impact on (1) the ability of the RNA-protein pairs to interact, and (2) on the performance of our proposed RPI prediction methods. Since the QQ string-based model shows a similar performance with the QSQS model, for simplicity, we only used the QQ model for this experiment. Further, the sequence length experiments are performed only on the RPI488 dataset, since this dataset was the most challenging for the string-based models, resulting in more prediction errors when compared with the other datasets (see the result tables in this section). Figure 5 shows a plot of the RPI prediction performance against protein sequence lengths and RNA sequence lengths. A quick observation shows that, most of the positive interaction pairs (Y’s and E’s) in the plots tended to occur at RNA lengths greater 200 nts, while most of the negative interaction pairs (N’s and F’s) seemed to occur mainly at RNA lengths below 200 nts. We could not discern any similar pattern for the impact of protein sequence lengths. However, in terms of the ability of our proposed string-based model to predict RPI, for RNA sequences greater than 200 nts, most of the prediction errors (red letters E or F) occurred at protein sequences less than 175 amino acids in length. For RNA sequences with lengths less than 200 nts, the protein sequence length did not seem to impact the ability of the method to predict the RPI. Since most of the positive pairs in the RPI488 dataset tend to have RNA sequence lengths above 200 nts, then best performance will be observed for protein sequence lengths above 175 amino acids.

### 5.5. Comparison with State-of-the-Art Methods

In order to evaluate our new models, we compared our work with three recent approaches reported in [3,10,16,23]. RPI-Pred [10] used sequences and secondary structures to build its method. It used both RNA sequence and secondary structure elements. It also used protein sequence and protein block as secondary structure representative. The other method is Muppirala et al. [3] which used only sequence information but they applied two different prediction methods: SVM and random forest. We will denote Muppirala et al.’s SVM method as RPISeq-SVM and their RF method as RPISeq-RF. IPMiner [23] used deep learning models, but only considered sequence information from the protein or RNA pairs, without using information about their secondary structures.

First we compared the results using RPI2241 dataset—see Table 5. Our feature-based method had an accuracy of 88.97%, performing better than RPI-Pred (84%) and RPISeq-SVM (87.1). The accuracy was very close to RPISeq-RF which performed the best, with an accuracy of 89.6%. Other performance measures followed the same pattern with RPISeq-RF performing better than other methods, followed closely by our feature-based method. Precision, recall and F-measure scores were as described in the table below. Interestingly enough, our feature-based method scored a better AUC (0.94) than RPISeq-RF (0.92), second to RPI-Seq-SVM (0.97). String-based method performed less than feature based method. Accuracy, precision, recall and F-measure were all very close to RPISeq-SVM and outperformed RPI-Pred as described below.

On the other hand, when testing against RPI369 dataset, the performance of both RPISeq-RF and RPISeq-SVM dropped to an accuracy of 76.2% and 72.8% respectively. RPI-Pred on the other hand, performed better, reporting accuracy of 92.0%. Both our feature-based and string-based methods outperformed all three methods, reporting an accuracy of 97.88% and 96.38% respectively. All other measures for our methods scored 0.95 or higher, better than all three methods. Table 6 compares the results when using RPI369 dataset.

To test the performance of the proposed approach on protein interaction with long non-coding RNAs (lncRNAs), we used the new dataset RPI488 reported in [23]. The dataset contains 243 interacting protein-lncRNA pairs, and 245 non-interacting pairs, for a total of 488 protein-lncRNA pairs. As indicated during our earlier analysis on impact of sequence lengths, this dataset was the most challenging of the four datasets evaluated in this work. Table 7 shows the results of our string-based approach (the QQ model) on this dataset, along with the results from other reported methods (taken from [23]). For this test, we used 10-fold cross validation, based on the top ranking *k*-mers with high richness values as generated from the RPI1807 dataset. We have reported the average result over the 10 folds. Accuracy ranged from 0.76 to 0.88, with an average of 0.82 over the 10 folds. As the table shows, the QQ string-based model did not perform as well as the other methods on this dataset. However, the results are still competitive, especially for accuracy. We have used richness *k*-mers, learned from RPI1807 dataset. Obviously, extracting the *k*-mers directly from the RPI488 would improve the performance. Also, we expect that the use of the full string-based models incorporating structure based information and the addition of the feature-based approach will lead to improved results on this dataset. 

Compared to other related work, we showed better consistency when using other datasets, such as RPI369, and RPI2241. Our string-based approach scored accuracy of at least 82% over different datasets. While feature-based method scored at least 88.97% when testing on RPI1807, RPI369, or RPI2441. We built our methods trying to cover as much RNA-protein pairs as possible. Results showed that our proposed approach can be reliable and used with different datasets without updating or changing the models or the selected features. 

## 6. Conclusions

The RNA-protein interaction (RPI) problem is an important problem in the analysis of molecular and cellular functions, since RNA-protein interaction has been implicated in many important cellular processes. The last few years have seen the discovery of more RNA and protein complexes, thus there is an increasing need for methods that can help us to determine the functions of such complexes, and the potential interaction between the entities involved. Given constraints due to cost and labor, and the increasingly large amounts of data involved, wet-lab experiments to determine RPI now require efficient and effective computational methods to help in the process. 

In this work, we introduce two different approaches to predict RPI. We exploited the size of RNA secondary structure elements as a significant descriptor for RNA secondary structures, and used the Ramachandran codes to represent protein secondary structures. This is the first attempt at using these two features for the RPI prediction problem. Further, we developed an innovative *k*-mer approach deploying powerful string data structures and algorithms on the problem of RPI prediction. The string-based approach maintained locality information which plays a crucial role in interaction between RNA and protein. Our results improved on previous approaches to RPI prediction, showing more consistency over different datasets and different types of RNA and protein. 

Further improvement could be obtained by finding better ways to integrate the sequence and structure information, and for intelligent fusion of the feature-based and string-based approaches. The string-based approach can also explore longer substrings (the *k*-mers), or perhaps inexact *k*-mers for possible performance improvement. Another approach could be in finding features from combined sequence and secondary structure data: we extracted *k*-mers from RNA sequences against protein sequences, and RNA secondary structures against protein secondary structures. Combining RNA sequence with secondary structure information and protein sequence with secondary structure information and then extracting *k*-mers could enhance the performance as it will be considering all available information jointly, rather than considering each one on its own.

## Figures and Tables

**Table 1:** Classes for RNA secondary structure lengths.

	Stem	Bulge	Loop	Internal Loop	Others (Not Classified)
Short	1–3	1–1	1–4	1–2	1–2
Medium	4–5	2–2	5–6	3–5	3–5
Long	≥6	≥3	≥7	≥6	≥6

**Table 2:** Performance for proposed methods using the RPI1807 dataset.

Metric	Feature-Based		String-Based	
	SVM	RF	SVM	RF
AUC	0.92	0.98	0.75	0.98
PRE	0.93	0.94	0.79	0.93
REC	0.92	0.94	0.74	0.93
FSC	0.92	0.94	0.73	0.93
ACC	92.94	94.68	74.00	93.35

**Table 3:** Results for the proposed string-based models using the RPI1807 dataset.

Metric	QSQS (SVM)	QSQS (RF)	QQ	SS	QS	SQ
#*k*-mers	7030	7030	4680	2350	3255	2955
AUC	0.75	0.98	0.93	0.66	0.64	0.95
PRE	0.79	0.93	0.93	0.79	0.78	0.91
REC	0.74	0.93	0.93	0.67	0.65	0.89
FSC	0.73	0.93	0.98	0.61	0.58	0.89
ACC (%)	74.00	93.35	93.19	66.98	65.15	89.29

**Table 4:** Potential influence of RNA SSE lengths on possible interaction between RNA-protein sequence pairs. Results are based on the RPI1807 dataset, using only the QQ string-based model. Red denotes RNA SSE types that are found mostly in negative pairs; Green denotes those that are found mostly in positive pairs; White denotes those with no specific dominance.

RNA SSE Type		Occurrences Within All Sequences				In Top 100 kmer Pairs (SS model)				In Top 100 kmer Pairs (QS model)				Dominant
(Based on Type, Length)	Code	PosPair	NegPair	Ratio	LogRatio	PosPair	NegPair	Ratio	LogRatio	PosPair	NegPair	Ratio	LogRatio	
Short Stem (SS)	A	333,596	331,862	1.005	0.008	77	161	0.481	−1.054	73	113	0.649	−0.623	Neg
Medium Stem (MS)	B	545,832	557,346	0.979	−0.030	129	69	1.857	0.893	106	67	1.574	0.654	Pos
Large Stem (LS)	C	702,214	735,912	0.954	−0.068	137	74	1.840	0.880	117	105	1.113	0.155	Pos
Short Loop (SL)	D	110,548	114,307	0.967	−0.048	22	10	2.091	1.064	0	5	0.167	−2.585	
Medium Loop (ML)	E	79,886	80,783	0.989	−0.016	16	5	2.833	1.503	19	5	3.333	1.737	Pos
Large Loop (LL)	F	68,838	68,450	1.006	0.008	5	3	1.500	0.585	5	36	0.162	−2.624	Neg
Small Internal Loop (SI)	G	197,645	197,748	0.999	−0.001	30	45	0.674	−0.569	34	48	0.714	−0.485	Neg
Medium Internal Loop (MI)	H	208,020	211,535	0.983	−0.024	22	30	0.742	−0.431	39	32	1.212	0.278	
Large Internal Loop (LI)	I	100,781	112,329	0.897	−0.157	20	17	1.167	0.222	35	10	3.273	1.710	Pos
Small Bulge (SB)	J	25,939	26,933	0.963	−0.054	10	14	0.733	−0.447	5	18	0.316	−1.663	Neg
Medium Bulge (MB)	K	20,190	20,984	0.962	−0.056	5	12	0.462	−1.115	12	19	0.650	−0.621	Neg
Large Bulge (LB)	L	95,362	99,365	0.960	−0.059	17	8	2.000	1.000	55	23	2.333	1.222	Pos
Small Others (SX)	M	2707	3007	0.900	−0.152	6	35	0.194	−2.363	0	0	1.000	0.000	
Medium Others (MX)	N	2679	2044	1.311	0.390	0	0	1.000	0.000	0	0	1.000	0.000	
Large Others (LX)	O	5866	6257	0.938	−0.093	4	0	5.000	2.322	0	0	1.000	0.000	
Totals		2,500,103	2,568,862			500	483			500	481			

**Table 5:** Comparative analysis of proposed string-based and feature-based models on RPI2241 dataset.

Metric	RPI2241					
	Feature-Based	String-Based	RPI-Pred [10]	RPISeq-SVM [3]	RPISeq-RF [3]	IPMiner [23]
AUC	0.94	0.92	0.89	0.97	0.92	0.91
PRE	0.89	0.86	0.88	0.87	0.89	0.84
REC	0.89	0.86	0.78	0.88	0.90	0.83
FSC	0.88	0.86	0.83	0.87	0.90	0.84
ACC	88.97	86.52	84.0	87.1	89.6	82.4

**Table 6:** Comparative analysis of proposed string-based and feature-based models on RPI369 dataset.

Metric	RPI369					
	Feature-Based	String-Based	RPI-Pred [10]	RPISeq-SVM [3]	RPISeq-RF [3]	IPMiner [23]
AUC	0.99	0.98	0.95	0.81	0.81	0.77
PRE	0.97	0.96	0.89	0.73	0.75	0.71
REC	0.97	0.96	0.89	0.73	0.78	0.74
FSC	0.97	0.96	0.89	0.73	0.77	0.73
ACC	97.88	96.38	92.0	72.8	76.2	75.2

**Table 7:** Comparative analysis of our proposed string-based QQ model on RPI488 protein-lncRNA dataset.

Metric	RPI488			
	QQ-Model	RPISeq-RF [3]	lncPro [16]	IPMiner [23]
PRE	0.77	0.93	0.91	0.89
REC	0.86	0.93	0.90	0.89
FSC	0.81	0.93	0.91	0.89
ACC	0.82	0.88	0.87	0.87

## Supplementary Materials

The following are available online: Tables for Top 100 *k*-mers for the four proposed string-based models. The following alphabets are used:

Protein Sequence:	{*A*, *I*, *Y, H*, *R*, *D*, *C*}
Protein Structure:	{*A*, *B*, *C*, *D*, *E*, *F*, *G*}
RNA Sequence:	{*A*, *U*, *C*, *G*, *X*}
RNA Structure:	{*A*, *B*, *C*, *D*, *E*, *F*, *G*, *H*, *I*, *J*, *K*, *L*, *M*, *N*, *O*}

## References

[1] Jankowsky E., Harris M.E. (2015). Specificity and nonspecificity in RNA–protein interactions. Nat. Rev. Mol. Cell Biol..

[2] Khalil A.M., Rinn J.L. (2011). RNA-protein interactions in human health and disease. Semin. Cell Dev. Biol..

[3] Muppirala U.K., Honavar V.G., Dobbs D. (2011). Predicting RNA-protein interactions using only sequence information. BMC Bioinform..

[4] Hofacker I.L. (2003). Vienna RNA secondary structure server. Nucleic Acids Res..

[5] Zuker M., Stiegler P. (1981). Optimal computer folding of large RNA sequences using thermodynamics and auxiliary information. Nucleic Acids Res..

[6] Lo W.C., Huang P.C., Chang C.H., Lyu P.C. (2007). Protein structural similarity search by Ramachandran codes. BMC Bioinform..

[7] Tan J., Adjeroh D. Text encoding for protein structure representation. Proceedings of the 45th Symposium on the Interface: Computing Science and Statistics.

[8] Hooft R.W., Sander C., Vriend G. (1997). Objectively judging the quality of a protein structure from a Ramachandran plot. CABIOS.

[9] De Brevern A.G., Etchebest C., Hazout S. (2000). Bayesian probabilistic approach for predicting backbone structures in terms of protein blocks. Proteins.

[10] Suresh V., Liu L., Adjeroh D., Zhou X. (2015). RPI-Pred: Predicting ncRNA-protein interaction using sequence and structural information. Nucleic Acids Res..

[11] Shen J., Zhang J., Luo X., Zhu W., Yu K., Chen K., Li Y., Jiang H. (2007). Predicting protein-protein interactions based only on sequences information. Proc. Natl. Acad. Sci. USA.

[12] Koh G.C., Porras P., Aranda B., Hermjakob H., Orchard S.E. (2012). Analyzing protein-protein interaction networks. J. Proteome Res..

[13] Epusz T., Yu H., Paccanaro A. (2012). Detecting overlapping protein complexes in protein-protein interaction networks. Nat. Methods.

[14] Reynolds C., Damerell D., Jones S. (2009). ProtorP: A protein-protein interaction analysis server. Bioinformatics.

[15] Wang Y., Chen X., Liu Z.P., Huang Q., Wang Y., Xu D., Zhang X.S., Chen R., Chen L. (2013). De novo prediction of RNA-protein interactions from sequence information. Mol. Biosyst..

[16] Lu Q., Ren S., Lu M., Zhang Y., Zhu D., Zhang X., Li T. (2013). Computational prediction of associations between long non-coding RNAs and proteins. BMC Genomics.

[17] Alipanahi B., Delong A., Weirauch M.T., Frey B.J. (2015). Predicting the sequence specificities of DNA- and RNA-binding proteins by deep learning. Nat. Biotechnol..

[18] Hassanzadeh H.R., Wang M.D. DeeperBind: Enhancing prediction of sequence specificities of DNA binding proteins. Proceedings of the IEEE International Conference on Bioinformatics and Biomedicine (BIBM).

[19] Pan X., Shen H.-B. (2017). RNA-protein binding motifs mining with a new hybrid deep learning based cross-domain knowledge integration approach. BMC Bioinform..

[20] Zhou J., Troyanskaya O.G. (2015). Predicting effects of noncoding variants with deep learning-based sequence model. Nat. Methods.

[21] Mamoshina P., Vieira A., Putin E., Zhavoronkov A. (2016). Applications of deep learning in biomedicine. Mol. Pharm..

[22] Jones W., Alasoo A., Fishman D., Parts L. (2017). Computational biology: deep learning. Emerg. Top. Life Sci..

[23] Pan X., Fan Y.X., Yan J., Shen H.B. (2016). IPMiner: Hidden ncRNA-protein interaction sequential pattern mining with stacked autoencoder for accurate computational prediction. BMC Genomics.

[24] Zhang S., Zhou J., Hu H., Gong H., Chen L., Cheng C., Zeng J. A deep learning framework for modeling structural features of RNA-binding protein targets. Nucleic Acids Res..

[25] Beal R., Adjeroh D. (2015). Efficient pattern matching for RNA secondary structures. Theor. Comput. Sci..

[26] Gusfield D. (1997). Algorithms on Strings, Trees, and Sequences: Computer Science and Computational Biology.

[27] Mattei E., Ausiello G., Ferre F., Helmer-Citterich M. (2014). A novel approach to represent and compare RNA secondary structures. Nucleic Acids Res..

[28] Rabani M., Kertesz M., Segal E. (2008). Computational prediction of RNA structural motifs involved in posttranscriptional regulatory processes. Proc. Natl. Acad. Sci. USA.

[29] Ramakrishnan C., Ramachandran G.N. (1966). Stereochemical criteria for polypeptide and protein chain conformations. III. Helical and hydrogen-bonded polypeptide chains. Biophys. J..

[30] Adjeroh D., Bell T.C., Mukherjee A. (2008). The Burrows-Wheeler Transform: Data Compression, Suffix Arrays, and Pattern Matching.

[31] Adjeroh D., Nan F. (2010). Suffix-Sorting via Shannon-Fano-Elias Codes. Algorithms.

[32] Lewis B.A., Walia R.R., Terribilini M., Ferguson J., Zheng C. (2011). PRIDB: A protein-RNA interface database. Nucleic Acids Res..

[33] Ma Y., Guo G. (2014). Support Vector Machines Applications.

[34] Breiman L. (2001). Random forests. Mach. Learn..

[35] Frank E., Hall M.A., Witten I.H. (2017). The WEKA Workbench. Online Appendix. Data Mining: Practical Machine Learning Tools and Techniques.

