# Supplementary material for: Feature-Based and String-Based Models for Predicting RNA-Protein Interaction
Source: Molecules. 2018 Mar 19;23(3):697. doi: 10.3390/molecules23030697 (PMC6017419; doi:10.3390/molecules23030697)
Supplement: Supplementary file 1 [file molecules-23-00697-s001.pdf]

Top Protein_RNA_k-mers: QQ model (Protein Sequence vs RNA Sequence

(Top 100 k-mers in Negative Pairs, but not in Positive Pairs)

Protein	RNA	Richness	#Positive pair	#Negative pair
ARHH	UGAU	-2.42651	0	266
YRDI	AGGC	-2.4133	0	258
YRDI	AUGC	-2.4133	0	258
YRDI	UGCA	-2.4133	0	258
YRDI	UUGC	-2.4133	0	258
YRDI	AAAC	-2.40654	0	254
YRDI	ACAC	-2.40654	0	254
YRDI	ACUC	-2.40654	0	254
YRDI	AUAG	-2.40654	0	254
YRDI	CAAG	-2.40654	0	254
YRDI	CUUG	-2.40654	0	254
YRDI	GAUA	-2.40654	0	254
YRDI	GUAU	-2.40654	0	254
YRDI	GUUA	-2.40654	0	254
YRDI	UAAG	-2.40654	0	254
YRDI	UAGA	-2.40654	0	254
YRDI	UCGA	-2.40654	0	254
YRDI	UGUA	-2.40654	0	254
YRDI	UUAA	-2.40654	0	254
YRDI	CAAA	-2.40312	0	252
ARHH	UCUA	-2.39967	0	250
YRDI	AAGA	-2.3962	0	248
YRDI	UAUG	-2.3927	0	246
DARH	CCCU	-2.38917	0	244
DARH	CACA	-2.3784	0	238
ARYIR	GGGUU	-2.37107	0	234
RYDD	ACGA	-2.37107	0	234
RYDD	CGUA	-2.37107	0	234
RYDD	GCGU	-2.37107	0	234
RYDD	UCGC	-2.37107	0	234
RYDD	AACA	-2.36736	0	232
RYDD	AACU	-2.36736	0	232
RYDD	ACAA	-2.36736	0	232
RYDD	ACAU	-2.36736	0	232
RYDD	ACCA	-2.36736	0	232
RYDD	ACUA	-2.36736	0	232
RYDD	ACUG	-2.36736	0	232
RYDD	ACUU	-2.36736	0	232
RYDD	AGAG	-2.36736	0	232
RYDD	AGUA	-2.36736	0	232
RYDD	AGUU	-2.36736	0	232
RYDD	AUAA	-2.36736	0	232
RYDD	AUAC	-2.36736	0	232
RYDD	AUGC	-2.36736	0	232
RYDD	AUGU	-2.36736	0	232
RYDD	AUUA	-2.36736	0	232
RYDD	CACU	-2.36736	0	232
RYDD	CAUA	-2.36736	0	232
RYDD	CUAU	-2.36736	0	232
RYDD	CUCA	-2.36736	0	232
RYDD	CUGG	-2.36736	0	232

(Top 100 k-mers in Positive Pairs, but not in Negative Pairs)

Protein	RNA	Richness	#Positive pair	#Negative pair
AIDA	UUCU	2.687529	486	0
AIDA	AUUG	2.680336	478	0
AIDA	AGUU	2.676694	474	0
AIDA	UCCU	2.652246	448	0
HAAY	GUGA	2.618048	414	0
HIDY	GGGG	2.61595	412	0
HIDY	GCCC	2.613842	410	0
HIDY	GGCC	2.608526	405	0
HIDY	ACCG	2.607455	404	0
HIDY	CCGC	2.607455	404	0
HIDY	CGAC	2.605305	402	0
HIDY	GACC	2.605305	402	0
HIDY	GGGC	2.604226	401	0
HIDY	AGCC	2.603144	400	0
HIDY	CGAA	2.603144	400	0
HIDY	CGAG	2.603144	400	0
HIDY	CUGC	2.603144	400	0
HIDY	GACG	2.603144	400	0
HIDY	GUCG	2.603144	400	0
HIDY	GGGA	2.60206	399	0
HIDY	CCGU	2.600973	398	0
HIDY	GUAG	2.600973	398	0
HIDY	UAGC	2.600973	398	0
HIDY	UGGG	2.600973	398	0
HIDY	CCCC	2.599883	397	0
HIDY	CCUG	2.598791	396	0
HIDY	GCGA	2.598791	396	0
HIDY	GCGC	2.598791	396	0
HIDY	ACGA	2.597695	395	0
HIDY	AGCG	2.596597	394	0
HIDY	AGGC	2.596597	394	0
HIDY	CUCC	2.596597	394	0
HIDY	GAAG	2.596597	394	0
HIDY	ACAG	2.595496	393	0
HIDY	CAGC	2.595496	393	0
HIDY	CGCG	2.595496	393	0
HIDY	GGAG	2.595496	393	0
HIDY	UGUG	2.595496	393	0
AIDA	UCUU	2.594393	392	0
HIDY	AACC	2.594393	392	0
HIDY	AAGG	2.594393	392	0
HIDY	ACGG	2.594393	392	0
HIDY	CGCC	2.594393	392	0
HIDY	CGUC	2.594393	392	0
HIDY	GCAA	2.594393	392	0
HIDY	UGCG	2.594393	392	0
HIDY	AAAG	2.592177	390	0
HIDY	CAGG	2.592177	390	0
HIDY	CCAG	2.592177	390	0
HIDY	CGUG	2.592177	390	0
HIDY	CUCA	2.592177	390	0

RYDD	GACU	-2.36736	0	232	HIDY	GAGG	2.592177	390	0
RYDD	GCAG	-2.36736	0	232	HIDY	GGAC	2.592177	390	0
RYDD	GUAA	-2.36736	0	232	HIDY	UCAG	2.592177	390	0
RYDD	GUCU	-2.36736	0	232	HIDY	UGCA	2.592177	390	0
RYDD	GUGU	-2.36736	0	232	HIDY	AGGA	2.58995	388	0
RYDD	GUUC	-2.36736	0	232	HIDY	AGGG	2.58995	388	0
RYDD	GUUU	-2.36736	0	232	HIDY	AGUG	2.58995	388	0
RYDD	UAAC	-2.36736	0	232	HIDY	AUCG	2.58995	388	0
RYDD	UAAU	-2.36736	0	232	HIDY	CUGG	2.58995	388	0
RYDD	UAUC	-2.36736	0	232	HIDY	GAAC	2.58995	388	0
RYDD	UAUG	-2.36736	0	232	HIDY	GAGC	2.58995	388	0
RYDD	UAUU	-2.36736	0	232	HIDY	GCCA	2.58995	388	0
RYDD	UCAA	-2.36736	0	232	HIDY	GCUC	2.58995	388	0
RYDD	UCAC	-2.36736	0	232	HIDY	GCUG	2.58995	388	0
RYDD	UCUG	-2.36736	0	232	HIDY	GGCU	2.58995	388	0
RYDD	UGCA	-2.36736	0	232	HIDY	GUGU	2.58995	388	0
RYDD	UGUA	-2.36736	0	232	HIDY	UACA	2.58995	388	0
RYDD	UUAC	-2.36736	0	232	HIDY	UAGG	2.58995	388	0
RYDD	UUAU	-2.36736	0	232	HIDY	UCGA	2.588832	387	0
RYDD	UUGA	-2.36736	0	232	HAAY	UGGU	2.587711	386	0
RYDD	UUUG	-2.36736	0	232	HIDY	ACCU	2.587711	386	0
YRDI	ACUU	-2.36736	0	232	HIDY	AGAC	2.587711	386	0
YRDI	CACA	-2.36736	0	232	HIDY	AGCU	2.587711	386	0
YRDI	CCAA	-2.36736	0	232	HIDY	AUAG	2.587711	386	0
YRDI	UGUU	-2.36736	0	232	HIDY	AUCA	2.587711	386	0
YRDI	UUAU	-2.36736	0	232	HIDY	CAGA	2.587711	386	0
ARYIR	CCUCG	-2.36361	0	230	HIDY	CUAG	2.587711	386	0
ARYIR	CGGGU	-2.36361	0	230	HIDY	GAGA	2.587711	386	0
RYDD	AAUG	-2.36361	0	230	HIDY	GAUC	2.587711	386	0
RYDD	ACAC	-2.36361	0	230	HIDY	GCUA	2.587711	386	0
RYDD	AUUC	-2.36361	0	230	HIDY	GGAA	2.587711	386	0
RYDD	CACA	-2.36361	0	230	HIDY	GAGU	2.586587	385	0
RYDD	GUUA	-2.36361	0	230	AIDA	AAAU	2.585461	384	0
RYDD	UACC	-2.36361	0	230	HIDY	AAGU	2.585461	384	0
YDDH	ACAA	-2.36361	0	230	HIDY	ACGC	2.585461	384	0
YDDH	ACUA	-2.36361	0	230	HIDY	ACUG	2.585461	384	0
YDDH	AGUU	-2.36361	0	230	HIDY	AGCA	2.585461	384	0
YDDH	AUUA	-2.36361	0	230	HIDY	AGUA	2.585461	384	0
YDDH	CCUU	-2.36361	0	230	HIDY	AUGG	2.585461	384	0
YDDH	GUCU	-2.36361	0	230	HIDY	AUGU	2.585461	384	0
YDDH	GUUU	-2.36361	0	230	HIDY	CAUG	2.585461	384	0
YDDH	UAAC	-2.36361	0	230	HIDY	CUGA	2.585461	384	0
YDDH	UACG	-2.36361	0	230	HIDY	GACA	2.585461	384	0
YDDH	UAUU	-2.36361	0	230	HIDY	GACU	2.585461	384	0
YDDH	UUAC	-2.36361	0	230	HIDY	GAUG	2.585461	384	0
YDDH	UUCU	-2.36361	0	230	HIDY	GCAG	2.585461	384	0
YDDH	UUGA	-2.36361	0	230	HIDY	GCGU	2.585461	384	0
YDDH	UUUG	-2.36361	0	230	HIDY	GUAA	2.585461	384	0
RYDD	CAAU	-2.35984	0	228	HIDY	UAGU	2.585461	384	0

Top Protein_RNA_k-mers: SQ model (Protein Structure vs RNA Sequence

(Top 100 k-mers in Negative Pairs, but not in Positive Pairs)

Protein	RNA	Richness	#Positive pair	#Negative pair
ARAYY	BJAAA	-2.44716	0	279
ARAYY	AAAAJ	-2.43775	0	273
ARAYY	EAAAB	-2.43616	0	272
ARAYY	JAAAA	-2.43457	0	271
ARAYY	KAAAJ	-2.43136	0	269
ARAYY	AGAAJ	-2.4216	0	263
ARAYY	GAAJA	-2.4216	0	263
ARAYY	GAJCC	-2.40824	0	255
RIYRR	BBGAJ	-2.39967	0	250
ARAYY	HAADD	-2.39445	0	247
ARAYY	HHAAD	-2.39445	0	247
ARAYY	HAGBB	-2.36736	0	232
ARAYY	HHAGB	-2.36736	0	232
ARAYY	GGAAC	-2.36361	0	230
ARAYY	GAGGC	-2.35218	0	224
RIYRR	LLAAG	-2.3483	0	222
RARRY	BAGBB	-2.34635	0	221
RARRY	BBAGB	-2.34635	0	221
ARAYY	BGAGG	-2.34635	0	221
RARRY	BGAJB	-2.34635	0	221
ARAYY	BGAJC	-2.34635	0	221
RARRY	CAAAD	-2.34635	0	221
ARAYY	FAAAG	-2.34635	0	221
ARAYY	GAAAF	-2.34635	0	221
RARRY	GGAAK	-2.34635	0	221
ARAYY	KAAAE	-2.34635	0	221
RARRY	KAAAE	-2.34635	0	221
ARAYY	JAAAE	-2.34242	0	219
ARAYY	LAACC	-2.32222	0	209
RARRY	LAACC	-2.32222	0	209
ARAYY	LLAAC	-2.32222	0	209
RARRY	LLAAC	-2.32222	0	209
RARRY	CJAAH	-2.32015	0	208
YAIRY	AAABB	-2.31597	0	206
YAIRY	AAAJB	-2.31597	0	206
YAIRY	AAFFF	-2.31597	0	206
YAIRY	AAJBB	-2.31597	0	206
YAIRY	AFFFF	-2.31597	0	206
YAIRY	BBAAG	-2.31597	0	206
YAIRY	BBBAA	-2.31597	0	206
YAIRY	BBBBK	-2.31597	0	206
YAIRY	BBBGC	-2.31597	0	206
YAIRY	BBBKK	-2.31597	0	206
YAIRY	BBGCC	-2.31597	0	206
YAIRY	BBGGC	-2.31597	0	206
YAIRY	BBKKA	-2.31597	0	206
YAIRY	BGCCC	-2.31597	0	206
YAIRY	BGGCC	-2.31597	0	206
YAIRY	BHHHA	-2.31597	0	206
YAIRY	BHHHC	-2.31597	0	206
YAIRY	CCCAA	-2.31597	0	206
YAIRY	CCCCF	-2.31597	0	206

(Top 100 k-mers in Positive Pairs, but not in Negative Pairs)

Protein	RNA	Richness	#Positive pair	#Negative pair
YRARI	EEEEE	2.418301	261	0
YRARI	HBBBB	2.418301	261	0
YRARI	HHBBB	2.418301	261	0
YRARI	HHHBB	2.418301	261	0
YRARI	IIIII	2.418301	261	0
YRARI	BBBBI	2.416641	260	0
YRARI	BBBII	2.416641	260	0
YRARI	BBIII	2.416641	260	0
YRARI	BIIII	2.416641	260	0
YRARI	CCCCH	2.416641	260	0
YRARI	CCCHH	2.416641	260	0
YRARI	CCHHH	2.416641	260	0
YRARI	HHHHB	2.416641	260	0
YRARI	JCCCC	2.416641	260	0
YRARI	AAEEE	2.414973	259	0
YRARI	AEEEE	2.414973	259	0
YRARI	AJCCC	2.414973	259	0
YRARI	BBBBL	2.414973	259	0
YRARI	BBBGB	2.414973	259	0
YRARI	BBBLL	2.414973	259	0
YRARI	BBGBB	2.414973	259	0
YRARI	BBLLL	2.414973	259	0
YRARI	BGBBB	2.414973	259	0
YRARI	CCCCA	2.414973	259	0
YRARI	CCCCI	2.414973	259	0
YRARI	CCCGA	2.414973	259	0
YRARI	CCCII	2.414973	259	0
YRARI	CCGAA	2.414973	259	0
YRARI	CCIII	2.414973	259	0
YRARI	CHHHH	2.414973	259	0
YRARI	CIIII	2.414973	259	0
YRARI	EEEAA	2.414973	259	0
YRARI	EEEEA	2.414973	259	0
YRARI	FFFFF	2.414973	259	0
YRARI	ICCCC	2.414973	259	0
YRARI	IICCC	2.414973	259	0
YRARI	IIICC	2.414973	259	0
YRARI	IIIIC	2.414973	259	0
YRARI	LBBBB	2.414973	259	0
YRARI	LLBBB	2.414973	259	0
YRARI	LLLBB	2.414973	259	0
YRARI	AAAAA	2.4133	258	0
YRARI	AAABB	2.4133	258	0
YRARI	AAAHH	2.4133	258	0
YRARI	AABBB	2.4133	258	0
YRARI	AAGGB	2.4133	258	0
YRARI	AAGGC	2.4133	258	0
YRARI	AAJAA	2.4133	258	0
YRARI	AALLL	2.4133	258	0
YRARI	ABBBB	2.4133	258	0
YRARI	AGAAA	2.4133	258	0
YRARI	AGGBB	2.4133	258	0

YAIRY	CCCCL	-2.31597	0	206
YAIRY	CCCFF	-2.31597	0	206
YAIRY	CCCGC	-2.31597	0	206
YAIRY	CCCLL	-2.31597	0	206
YAIRY	CCFFF	-2.31597	0	206
YAIRY	CCGCC	-2.31597	0	206
YAIRY	CCLLL	-2.31597	0	206
YAIRY	CFFFF	-2.31597	0	206
YAIRY	CGCCC	-2.31597	0	206
YAIRY	CLLLL	-2.31597	0	206
YAIRY	FCCCC	-2.31597	0	206
YAIRY	FFCCC	-2.31597	0	206
YAIRY	FFFAA	-2.31597	0	206
YAIRY	FFFCC	-2.31597	0	206
YAIRY	FFFFA	-2.31597	0	206
YAIRY	FFFFC	-2.31597	0	206
YAIRY	GAAAH	-2.31597	0	206
YAIRY	HCCCC	-2.31597	0	206
YAIRY	HHCCC	-2.31597	0	206
YAIRY	HHHCC	-2.31597	0	206
YAIRY	HHHHC	-2.31597	0	206
YAIRY	ICCCC	-2.31597	0	206
YAIRY	IICCC	-2.31597	0	206
YAIRY	IIICC	-2.31597	0	206
YAIRY	IIIIC	-2.31597	0	206
YAIRY	KKAAA	-2.31597	0	206
YAIRY	LLLAA	-2.31597	0	206
YAIRY	AAACC	-2.31387	0	205
YAIRY	AAJCC	-2.31387	0	205
YAIRY	BJAAA	-2.31387	0	205
YAIRY	CCAAA	-2.31387	0	205
YAIRY	CCGGB	-2.31387	0	205
YAIRY	CGGBB	-2.31387	0	205
YAIRY	GAAAC	-2.31387	0	205
YAIRY	GGAAG	-2.31387	0	205
YAIRY	JAAAG	-2.31175	0	204
YAIRY	AJAAA	-2.30963	0	203
ARAYY	HAGGB	-2.3075	0	202
YAIRY	AHHHC	-2.30535	0	201
YAIRY	CHHHC	-2.30535	0	201
YAIRY	KBBBB	-2.30535	0	201
YAIRY	KKBBB	-2.30535	0	201
YAIRY	BBGAG	-2.3032	0	200
YAIRY	BBKKB	-2.3032	0	200
YAIRY	BKKBB	-2.3032	0	200
YAIRY	HAAAD	-2.3032	0	200
YAIRY	LLAAA	-2.3032	0	200
YAIRY	KCCCC	-2.30103	0	199

YRARI	AGGCC	2.4133	258	0
YRARI	AHHHB	2.4133	258	0
YRARI	ALLLL	2.4133	258	0
YRARI	BBBBK	2.4133	258	0
YRARI	BBBKK	2.4133	258	0
YRARI	BBGGA	2.4133	258	0
YRARI	BGGAA	2.4133	258	0
YRARI	BLLLL	2.4133	258	0
YRARI	CCAAA	2.4133	258	0
YRARI	CCCAA	2.4133	258	0
YRARI	CCCGC	2.4133	258	0
YRARI	CCGCC	2.4133	258	0
YRARI	CGCCC	2.4133	258	0
YRARI	GGCCC	2.4133	258	0
YRARI	HHHHH	2.4133	258	0
YRARI	KBBBB	2.4133	258	0
YRARI	KKAAA	2.4133	258	0
YRARI	KKBBB	2.4133	258	0
YRARI	LCCCC	2.4133	258	0
YRARI	LLCCC	2.4133	258	0
YRARI	LLLAA	2.4133	258	0
YRARI	LLLCC	2.4133	258	0
YRARI	LLLLB	2.4133	258	0
YRARI	LLLLC	2.4133	258	0
YRARI	LLLLL	2.4133	258	0
ARAAA	BBGAJ	2.41162	257	0
AYRAR	BBKKB	2.41162	257	0
YRARI	BHHHC	2.41162	257	0
AYRAR	BKKBB	2.41162	257	0
YRARI	CCGGC	2.41162	257	0
YRARI	CGGCC	2.41162	257	0
YRARI	GAAGB	2.41162	257	0
YRARI	GGAAG	2.41162	257	0
YRARI	BBBBC	2.409933	256	0
YRARI	BBBCC	2.409933	256	0
YRARI	BBCCC	2.409933	256	0
YRARI	BCCCC	2.409933	256	0
YRARI	CCCCL	2.409933	256	0
YRARI	CCCLL	2.409933	256	0
YRARI	CCLLL	2.409933	256	0
YRARI	CHHHB	2.409933	256	0
YRARI	AAACC	2.40824	255	0
YRARI	AACCC	2.40824	255	0
YRARI	ACCCC	2.40824	255	0
YRARI	AJAAA	2.40824	255	0
RAAAA	BBBAG	2.40824	255	0
YRARI	CHHHA	2.40824	255	0
YRARI	CLLLL	2.40824	255	0

Top Protein_RNA_k-mers : SQ model (Protein Structure vs RNA Sequence)

(Top 100 k-mers in Negative Pairs, but not in Positive Pairs)

Protein	RNA	Richness	#Positive pair	#Negative pair
CADDD	AGUAU	-1.79934	0	62
CADDD	UACAU	-1.78533	0	60
BFADD	ACCUC	-1.65321	0	44
BFADD	CAAUC	-1.65321	0	44
BFADD	CACGA	-1.65321	0	44
BFADD	CAUCC	-1.65321	0	44
BFADD	CCAAU	-1.65321	0	44
BFADD	UUCGC	-1.65321	0	44
BFADD	CACAU	-1.60206	0	39
BFADD	CAGAC	-1.60206	0	39
BFADD	CGUAU	-1.60206	0	39
BFADD	GCUAU	-1.60206	0	39
BFADD	UAUGC	-1.60206	0	39
ACADD	AGUAU	-1.57978	0	37
ACADD	UACAU	-1.57978	0	37
AECCE	AGUAU	-1.5563	0	35
AECCE	CAUAC	-1.5563	0	35
ECCAD	UCCUC	-1.54407	0	34
AECCE	AAUAA	-1.53148	0	33
AECCE	CAAUA	-1.53148	0	33
AECCE	CGUUU	-1.53148	0	33
AECCE	CUACU	-1.53148	0	33
AECCE	UACAU	-1.53148	0	33
AECCE	UCGUU	-1.53148	0	33
AECCE	UUCUU	-1.53148	0	33
BAEEC	AUCAC	-1.53148	0	33
ECCAD	AAUGU	-1.53148	0	33
ECCAD	ACAUA	-1.53148	0	33
ECCAD	AUACA	-1.53148	0	33
ECCAD	AUAGU	-1.53148	0	33
ECCAD	AUAUC	-1.53148	0	33
ECCAD	CAGAC	-1.53148	0	33
ECCAD	CUAUG	-1.53148	0	33
ECCAD	CUCAU	-1.53148	0	33
ECCAD	CUGCU	-1.53148	0	33
ECCAD	GAUAU	-1.53148	0	33
ECCAD	GCUAU	-1.53148	0	33
ECCAD	GUAUC	-1.53148	0	33
ECCAD	UACAG	-1.53148	0	33
ECCAD	UAUCG	-1.53148	0	33
ECCAD	UAUCU	-1.53148	0	33
ECCAD	UAUGC	-1.53148	0	33
ECCAD	UAUGG	-1.53148	0	33
ECCAD	UCAUA	-1.53148	0	33
ECCAD	UCCAU	-1.53148	0	33
ECCAD	UCUGU	-1.53148	0	33
ECCAD	UGCUU	-1.53148	0	33
ECCAD	UUAGC	-1.53148	0	33
ECCAD	UUCCA	-1.53148	0	33
BAEEC	CACUU	-1.51851	0	32
ECCAD	ACAGA	-1.51851	0	32

(Top 100 k-mers in Positive Pairs, but not in Negative Pairs)

Protein	RNA	Richness	#Positive pair	#Negative pair
FAFFE	UCAUA	1.869232	73	0
FAFFE	GUCUA	1.863323	72	0
FABAF	CAACA	1.851258	70	0
FAFFE	CAUUC	1.799341	62	0
FECFC	AACUA	1.799341	62	0
FECFC	AUAAC	1.799341	62	0
FECFC	AUAGG	1.799341	62	0
FECFC	CCUCG	1.799341	62	0
FECFC	CCUGC	1.799341	62	0
FECFC	GACAG	1.799341	62	0
FECFC	GGCAU	1.799341	62	0
FECFC	UACCU	1.799341	62	0
FECFC	UAUAG	1.799341	62	0
FECFC	AAAAC	1.792392	61	0
FECFC	AAGAU	1.792392	61	0
FECFC	AAUGA	1.792392	61	0
FECFC	ACAGC	1.792392	61	0
FECFC	ACCUC	1.792392	61	0
FECFC	AGAAU	1.792392	61	0
FECFC	AGUGG	1.792392	61	0
FECFC	AUGAC	1.792392	61	0
FECFC	CAAGA	1.792392	61	0
FECFC	CCAAU	1.792392	61	0
FECFC	CCGUA	1.792392	61	0
FECFC	CGAAC	1.792392	61	0
FECFC	CGAAU	1.792392	61	0
FECFC	CGAGU	1.792392	61	0
FECFC	CGAUC	1.792392	61	0
FECFC	CGUAG	1.792392	61	0
FECFC	CUCGA	1.792392	61	0
FECFC	CUGUC	1.792392	61	0
FECFC	GAAAA	1.792392	61	0
FECFC	GAACA	1.792392	61	0
FECFC	GACUC	1.792392	61	0
FECFC	GAGAA	1.792392	61	0
FECFC	GAGUG	1.792392	61	0
FECFC	GCAUA	1.792392	61	0
FECFC	GGUUG	1.792392	61	0
FECFC	GUCCU	1.792392	61	0
FECFC	UAACC	1.792392	61	0
FECFC	UAGUG	1.792392	61	0
FECFC	UCAAG	1.792392	61	0
FECFC	UCGAC	1.792392	61	0
FECFC	UCGAU	1.792392	61	0
FECFC	UGUCU	1.792392	61	0
FECFC	AAAAU	1.78533	60	0
FECFC	AAACA	1.78533	60	0
FECFC	AAAGU	1.78533	60	0
FECFC	AAAUA	1.78533	60	0
FECFC	AAAUC	1.78533	60	0
FECFC	AACAC	1.78533	60	0

ECCAD	AGUAU	-1.51851	0	32
ECCAD	AUGUU	-1.51851	0	32
ECCAD	AUUGC	-1.51851	0	32
ECCAD	CAUAC	-1.51851	0	32
ECCAD	CGUUU	-1.51851	0	32
ECCAD	UUGCU	-1.51851	0	32
ECCAD	UUGUU	-1.51851	0	32
BAEEC	AACCA	-1.50515	0	31
BAEEC	AUAUC	-1.50515	0	31
BAEEC	AUCCA	-1.50515	0	31
BAEEC	CACAU	-1.50515	0	31
BAEEC	CAGAC	-1.50515	0	31
BAEEC	CAUAG	-1.50515	0	31
BAEEC	CCAUA	-1.50515	0	31
BAEEC	CUAUG	-1.50515	0	31
BAEEC	GCUAU	-1.50515	0	31
BAEEC	UACAG	-1.50515	0	31
BAEEC	UCAUA	-1.50515	0	31
ECCAD	AAUCA	-1.50515	0	31
ECCAD	AAUUA	-1.50515	0	31
ECCAD	ACAUU	-1.50515	0	31
ECCAD	ACUAA	-1.50515	0	31
ECCAD	ACUUA	-1.50515	0	31
ECCAD	AGAUU	-1.50515	0	31
ECCAD	AUACU	-1.50515	0	31
ECCAD	AUUUC	-1.50515	0	31
ECCAD	CACAU	-1.50515	0	31
ECCAD	CAUAU	-1.50515	0	31
ECCAD	CGUAU	-1.50515	0	31
ECCAD	GAUUU	-1.50515	0	31
ECCAD	GUCAU	-1.50515	0	31
ECCAD	UAUGU	-1.50515	0	31
ECCAD	UAUUA	-1.50515	0	31
ECCAD	UCAUC	-1.50515	0	31
ECCAD	UCUUA	-1.50515	0	31
ECCAD	UGUUA	-1.50515	0	31
ECCAD	UUAAC	-1.50515	0	31
ECCAD	UUCUG	-1.50515	0	31
ECCAD	UUGCA	-1.50515	0	31
BAEEC	AUUGC	-1.49136	0	30
BAEEC	CUUCA	-1.49136	0	30
BAEEC	UCGUU	-1.49136	0	30
BAEEC	UUGCU	-1.49136	0	30
CADDA	CGUUU	-1.49136	0	30
DBAEE	ACAUG	-1.49136	0	30
DBAEE	ACCUU	-1.49136	0	30
DBAEE	AGCAA	-1.49136	0	30
DBAEE	AGUCG	-1.49136	0	30
DBAEE	AUCAC	-1.49136	0	30

FECFC	AACAG	1.78533	60	0
FECFC	AACAU	1.78533	60	0
FECFC	AACGU	1.78533	60	0
FECFC	AACUG	1.78533	60	0
FECFC	AACUU	1.78533	60	0
FECFC	AAGUA	1.78533	60	0
FECFC	AAGUG	1.78533	60	0
FECFC	AAGUU	1.78533	60	0
FECFC	AAUAU	1.78533	60	0
FECFC	ACCCA	1.78533	60	0
FECFC	ACCCU	1.78533	60	0
FECFC	ACCGA	1.78533	60	0
FECFC	ACGCA	1.78533	60	0
FECFC	ACUGU	1.78533	60	0
FECFC	AGAAA	1.78533	60	0
FECFC	AGAGA	1.78533	60	0
FECFC	AGAGC	1.78533	60	0
FECFC	AGAUG	1.78533	60	0
FECFC	AGCUU	1.78533	60	0
FECFC	AGGAC	1.78533	60	0
FECFC	AGGUU	1.78533	60	0
FECFC	AGUCU	1.78533	60	0
FECFC	AGUGA	1.78533	60	0
FECFC	AGUGC	1.78533	60	0
FECFC	AUAAG	1.78533	60	0
FECFC	AUAUU	1.78533	60	0
FECFC	AUCCG	1.78533	60	0
FECFC	AUCGA	1.78533	60	0
FECFC	AUCGG	1.78533	60	0
FECFC	AUCUA	1.78533	60	0
FECFC	AUCUC	1.78533	60	0
FECFC	AUCUG	1.78533	60	0
FECFC	AUGAG	1.78533	60	0
FECFC	AUGAU	1.78533	60	0
FECFC	AUGGA	1.78533	60	0
FECFC	AUGUC	1.78533	60	0
FECFC	AUUAA	1.78533	60	0
FECFC	CAAUC	1.78533	60	0
FECFC	CACGA	1.78533	60	0
FECFC	CAGAA	1.78533	60	0
FECFC	CAGAG	1.78533	60	0
FECFC	CAGGA	1.78533	60	0
FECFC	CAGUA	1.78533	60	0
FECFC	CAGUG	1.78533	60	0
FECFC	CAUCU	1.78533	60	0
FECFC	CCAGA	1.78533	60	0
FECFC	CCCUC	1.78533	60	0
FECFC	CCCUG	1.78533	60	0
FECFC	CCUAU	1.78533	60	0

Top Protein_RNA_k-mers: SS model (Protein Structure vs RNA Structure)

(Top 100 k-mers in Negative Pairs, but not in Positive Pairs)

Protein	RNA	Richness	#Positive pair	#Negative pair
CADDD	CCMMB	-1.79934	0	62
CADDD	CMMBB	-1.79934	0	62
CADDD	HAAAF	-1.78533	0	60
CADDD	CCGAH	-1.77815	0	59
CADDD	CGAHH	-1.77815	0	59
CADDD	BAACC	-1.76343	0	57
CADDD	BBAAC	-1.76343	0	57
CADDD	CJAAL	-1.76343	0	57
CADDD	DAAAK	-1.76343	0	57
CADDD	KAAAI	-1.76343	0	57
CADDD	AAGAJ	-1.74819	0	55
CADDD	AGAJB	-1.74819	0	55
CADDD	BAGAA	-1.74819	0	55
CADDD	BBAGA	-1.74819	0	55
CADDD	CGAAE	-1.74819	0	55
CDDDD	BGGAD	-1.74819	0	55
CDDDD	DAGGB	-1.74819	0	55
CDDD	CMMB	-1.73239	0	53
CDDD	BAAC	-1.716	0	51
CDDDD	CCMMB	-1.716	0	51
CDDDD	CMMBB	-1.716	0	51
CDDD	BBMC	-1.70757	0	50
CDDD	BMCC	-1.70757	0	50
CDDDD	BAACC	-1.69897	0	49
CDDDD	BBAAC	-1.69897	0	49
CDDDD	CJAAL	-1.69897	0	49
CDDDD	BBBMC	-1.6902	0	48
CDDDD	BBMCC	-1.6902	0	48
CDDDD	BMCCC	-1.6902	0	48
CDDDD	KAAAI	-1.6902	0	48
CDDDD	AAGAJ	-1.68124	0	47
CDDDD	AGAJB	-1.68124	0	47
CDDDD	BAGAA	-1.68124	0	47
CDDDD	BBAGA	-1.68124	0	47
CDDDD	CGAAE	-1.68124	0	47
BFADD	EAAGG	-1.65321	0	44
BFADD	GAAAL	-1.65321	0	44
BFADD	HAAAI	-1.65321	0	44
BFADD	HAGCC	-1.65321	0	44
BFADD	HHAGC	-1.65321	0	44
BFADD	JAAAH	-1.65321	0	44
BFADD	BGGAD	-1.64345	0	43
BFADD	CAAGG	-1.64345	0	43
BFADD	CCAAG	-1.64345	0	43
BFADD	DAAHH	-1.64345	0	43
BFADD	DAGGB	-1.64345	0	43
BFADD	DDAAH	-1.64345	0	43
BFADD	GGAAJ	-1.64345	0	43
BFADD	HAABB	-1.64345	0	43
BFADD	HAAJC	-1.64345	0	43
BFADD	HHAAB	-1.64345	0	43
CFDD	CGAH	-1.64345	0	43

(Top 100 k-mers in Positive Pairs, but not in Negative Pairs)

Protein	RNA	Richness	#Positive pair	#Negative pair
DDCD	OOOO	1.959041	90	0
DDCD	BBBM	1.939519	86	0
DDCD	MBBB	1.939519	86	0
DDCD	MMBB	1.939519	86	0
DDCD	IAAA	1.934498	85	0
DCDF	BBBB	1.892095	77	0
DCDF	BBBD	1.892095	77	0
DCDF	BBDD	1.892095	77	0
DCDF	BDDD	1.892095	77	0
DCDF	DBBB	1.892095	77	0
DCDF	DDBB	1.892095	77	0
DCDF	DDDB	1.892095	77	0
DCDF	BBBE	1.886491	76	0
DCDF	BBBH	1.886491	76	0
DCDF	BBBJ	1.886491	76	0
DCDF	BBBL	1.886491	76	0
DCDF	BBEE	1.886491	76	0
DCDF	BBHH	1.886491	76	0
DCDF	BBLL	1.886491	76	0
DCDF	BEEE	1.886491	76	0
DCDF	BHHH	1.886491	76	0
DCDF	BLLL	1.886491	76	0
DCDF	CCCC	1.886491	76	0
DCDF	CCCI	1.886491	76	0
DCDF	CCII	1.886491	76	0
DCDF	CIII	1.886491	76	0
DCDF	DDDD	1.886491	76	0
DCDF	EBBB	1.886491	76	0
DCDF	EEBB	1.886491	76	0
DCDF	EEEB	1.886491	76	0
DCDF	EEEE	1.886491	76	0
DCDF	HBBB	1.886491	76	0
DCDF	HHBB	1.886491	76	0
DCDF	HHHB	1.886491	76	0
DCDF	HHHH	1.886491	76	0
DCDF	IBBB	1.886491	76	0
DCDF	IIBB	1.886491	76	0
DCDF	IIIB	1.886491	76	0
DCDF	IIII	1.886491	76	0
DCDF	JCCC	1.886491	76	0
DCDF	LBBB	1.886491	76	0
DCDF	LLBB	1.886491	76	0
DCDF	LLLB	1.886491	76	0
DDCD	BBMM	1.886491	76	0
DCDF	AAAA	1.880814	75	0
DCDF	AAAC	1.880814	75	0
DCDF	AAAD	1.880814	75	0
DCDF	AAAG	1.880814	75	0
DCDF	AAAH	1.880814	75	0
DCDF	AAAJ	1.880814	75	0
DCDF	AABB	1.880814	75	0
DCDF	AACC	1.880814	75	0

BFADD	CAAHH	-1.63347	0	42	DCDF	AADD	1.880814	75	0
BFADD	CCAAH	-1.63347	0	42	DCDF	AAFF	1.880814	75	0
BFADD	CLLLC	-1.61278	0	40	DCDF	AAGA	1.880814	75	0
CCAD	CMMB	-1.61278	0	40	DCDF	AAGB	1.880814	75	0
BFAD	AGAJ	-1.60206	0	39	DCDF	AAGC	1.880814	75	0
BFAD	BAAC	-1.60206	0	39	DCDF	AAGG	1.880814	75	0
BFAD	BAGA	-1.60206	0	39	DCDF	AAHH	1.880814	75	0
BFAD	BBMC	-1.60206	0	39	DCDF	AAJB	1.880814	75	0
BFAD	BMCC	-1.60206	0	39	DCDF	AAJC	1.880814	75	0
BFAD	CMMB	-1.60206	0	39	DCDF	AAKK	1.880814	75	0
BFADD	FAAAI	-1.60206	0	39	DCDF	AALL	1.880814	75	0
BFADD	IAAAH	-1.60206	0	39	DCDF	ABBB	1.880814	75	0
BFADD	IAAGB	-1.60206	0	39	DCDF	ACCC	1.880814	75	0
BFAD	HAAI	-1.60206	0	39	DCDF	ADDD	1.880814	75	0
BFAD	KAAI	-1.60206	0	39	DCDF	AFFF	1.880814	75	0
ACADD	CCMMB	-1.59106	0	38	DCDF	AGAA	1.880814	75	0
ACADD	CMMBB	-1.59106	0	38	DCDF	AGBB	1.880814	75	0
BAEE	GAAI	-1.59106	0	38	DCDF	AGCC	1.880814	75	0
BFADD	AAGAJ	-1.59106	0	38	DCDF	AGGA	1.880814	75	0
BFADD	AGAJB	-1.59106	0	38	DCDF	AGGB	1.880814	75	0
BFADD	BAACC	-1.59106	0	38	DCDF	AGGC	1.880814	75	0
BFADD	BAGAA	-1.59106	0	38	DCDF	AHHH	1.880814	75	0
BFADD	BBAAC	-1.59106	0	38	DCDF	AJAA	1.880814	75	0
BFADD	BBAGA	-1.59106	0	38	DCDF	AJBB	1.880814	75	0
BFADD	BBBMC	-1.59106	0	38	DCDF	AJCC	1.880814	75	0
BFADD	BBMCC	-1.59106	0	38	DCDF	ALLL	1.880814	75	0
BFADD	BMCCC	-1.59106	0	38	DCDF	BBAA	1.880814	75	0
BFADD	CCMMB	-1.59106	0	38	DCDF	BBBA	1.880814	75	0
BFADD	CGAAE	-1.59106	0	38	DCDF	BBBC	1.880814	75	0
BFADD	CJAAL	-1.59106	0	38	DCDF	BBBG	1.880814	75	0
BFADD	CMMBB	-1.59106	0	38	DCDF	BBBI	1.880814	75	0
BFADD	HAAII	-1.59106	0	38	DCDF	BBBK	1.880814	75	0
BFADD	HHAAI	-1.59106	0	38	DCDF	BBCC	1.880814	75	0
BFADD	KAAAI	-1.59106	0	38	DCDF	BBGA	1.880814	75	0
BFADD	KAAII	-1.59106	0	38	DCDF	BBGB	1.880814	75	0
BFADD	KKAAI	-1.59106	0	38	DCDF	BBGC	1.880814	75	0
AAED	BBMC	-1.57978	0	37	DCDF	BBGG	1.880814	75	0
AAED	BMCC	-1.57978	0	37	DCDF	BBII	1.880814	75	0
ACADD	KAAAI	-1.57978	0	37	DCDF	BBJA	1.880814	75	0
AAED	CGAH	-1.5682	0	36	DCDF	BBJC	1.880814	75	0
ACADD	DAAAK	-1.5682	0	36	DCDF	BBKK	1.880814	75	0
BBDD	JAAL	-1.5682	0	36	DCDF	BCCC	1.880814	75	0
ACADD	CCGAH	-1.5563	0	35	DCDF	BGAA	1.880814	75	0
ACADD	CGAAE	-1.5563	0	35	DCDF	BGBB	1.880814	75	0
ACADD	CGAHH	-1.5563	0	35	DCDF	BGCC	1.880814	75	0
AECCE	BAAGA	-1.5563	0	35	DCDF	BGGA	1.880814	75	0
AECCE	DAAAK	-1.5563	0	35	DCDF	BGGB	1.880814	75	0
AECCE	HAAAF	-1.5563	0	35	DCDF	BGGC	1.880814	75	0
